# Effect of Immediate and Delayed Finishing and Polishing on the Surface Roughness and Microhardness of Alkasite and Giomer

**DOI:** 10.4317/jced.63417

**Published:** 2025-11-30

**Authors:** Hanan A. Soliman, John Comisi, Dina Abdelaziz

**Affiliations:** 1 Conservative Dentistry Department, Faculty of Dentistry, Kafrelsheikh University, Kafrelsheikh, Egypt; 2 Department of General Dentistry, The Dental College of Georgia, Augusta University, USA; 3 Department of Dental Biomaterials, Faculty of Dentistry, Mansoura University, Egypt

## Abstract

**Background:**

Finishing and polishing (F/P) procedures are essential for optimizing resin-based restoration esthetics, wear resistance, and surface quality. Bioactive materials like Alkasite and Giomer have attracted attention for their potential clinical benefits, although limited evidence exists regarding the impact of immediate versus delayed F/P protocols on their surface properties. This study assessed how F/P timing and systems influence the surface roughness and microhardness of these materials.

**Material and Methods:**

One hundred cylindrical specimens (n=50 per material) were prepared and divided into five subgroups: control (Mylar strip), immediate Sof-Lex, delayed Sof-Lex, immediate Politip, and delayed Politip. Surface roughness was measured using a profilometer, surface morphology was examined with SEM, and microhardness was tested with a Vickers hardness testing. Two-way ANOVA and Tukey's post hoc test were used to analyze the data (p &lt; 0.05).

**Results:**

Control groups exhibited the smoothest surfaces, with Giomer showing significantly lower roughness values than Alkasite across all subgroups. In Giomer, Sof-Lex produced the lowest Ra values, while Alkasite showed no significant differences between protocols. Microhardness results revealed no significant variation between materials regardless of F/P system or timing.

**Conclusions:**

The timing of F/P procedures did not significantly affect the surface roughness or microhardness of Alkasite or Giomer.

## Introduction

Resin composites have been widely used in conservative dentistry because of their key benefits, including excellent aesthetics, minimal tooth preparation, and effective preservation of dental structures. (1 , 2) However, challenges such as technique sensitivity and the time-consuming incremental technique have driven the development of advanced resin-based restoratives. Recent research has focused on creating aesthetically pleasing materials that also inhibit caries progression and promote remineralization (3). Giomer is regarded as a true hybrid material, combining the properties of glass ionomer and composite resin. It contains pre-reacted glass-ionomer filler particles dispersed within a resin matrix, providing the fluoride-releasing capability of glass ionomers along with the esthetic qualities, mechanical strength, and ease of handling of composite resins (4). Recently, alkasite-based materials (Cention N) have been introduced for restorative dental procedures. The term "alkasite" refers to the inclusion of alkaline glass filler particles that release calcium and fluoride ions, which promote remineralization and protect against demineralization during acidic challenges. These materials also release hydroxide ions to help regulate pH (5). Cention N is mainly self-curing, with the option of light curing due to the presence of acyl phosphine oxide and Ivocerin photoinitiators (6). It is suitable for restorations in stress-bearing regions, meeting the ISO 4049 requirement of at least 80 MPa flexural strength in polymer-based restoratives. Both Giomer and Alkasite are appropriate for managing carious lesions because of their fluoride-releasing properties, which aid in caries prevention (7 , 8). Surface smoothness is considered one of the most important factors for achieving both effective and long-lasting tooth-colored restorations. Insufficient finishing and polishing (F/P) can increase the surface roughness of restorations, which in turn encourages bacterial adhesion, secondary caries development, and may weaken the restoration's reparability (8 , 9). According to Bollen, surface roughness values above 0.2 µm are generally known to promote bacterial adhesion (10). An appropriate finishing and polishing (F/P) protocol is crucial for improving the aesthetic quality and longevity of dental restorations. The finishing phase includes removing excess material and shaping the restoration to replicate natural anatomical features. This is followed by polishing, which produces a smooth, high-gloss surface resembling natural enamel's texture (11). Various F/P systems are available, including rubber cups, carbide burs, and silicone or aluminum-oxide discs, which can be used in one-step, two-step, or multiple-step polishing methods (12). However, the effectiveness of F/P procedures in tooth-colored materials depends on the geometry, concentration, and size of fillers. Both the choice of F/P systems and the properties of fillers, particularly their size, play a significant role in determining the final surface roughness (13). Hardness is another critical factor closely linked to surface properties. Similar to surface roughness, surface hardness plays a vital role in the long-term performance of esthetic restorative materials. It reflects the wear resistance of the material and its ability to withstand abrasion from opposing teeth or restorative materialsm (14). Since finishing and polishing affect both surface roughness and hardness, optimizing these processes is crucial for maintaining the functional durability and aesthetic quality of restorative materials (1). The literature shows inconsistent results regarding the best timing for composite restoration finishing and polishing (F/P). Manufacturers often recommend performing F/P soon after placement, and some studies support immediate polishing to decrease the number of clinical visits (15). However, Amir Ghasemi et al. found that immediate F/P resulted in poorer surface properties, whereas delaying the process significantly increased the material's hardness (16). Similarly, another study suggested a 24-hour delay to achieve better clinical results (17). This improvement may be due to heat-related effects that occur when finishing and polishing are done before the composite fully polymerizes. Such thermal effects, including resin matrix smearing and localized hotspot formation, can harm the restoration's surface quality (18). So far, the role of polishing timing in affecting the surface roughness of Alkasite-based restorations has not been specifically studied. Therefore, this study aimed to examine how immediate versus delayed finishing and polishing influence the surface properties of Alkasite restorations, using aluminum-oxide discs and rubber polishing points, with Giomer-based materials included for comparison. The study tested two null hypotheses: the first was that the timing of the F/P procedure (immediate versus delayed) would have no significant impact on surface roughness or microhardness; the second was that the type of F/P tools used would not significantly affect these surface properties.

## Material and Methods

- Sample size calculation The required sample size was estimated using G*Power software with an effect size of 0.29, a power of 80%, and a significance level of 0.05 (19). The calculated sample size was 96 specimens. This was increased to a total of 100 specimens, with 10 specimens in each subgroup. - Specimen preparation This study used two different materials: a Giomer material (Group A) (Beautifil II, Shofu Inc., Kyoto, Japan) and an alkasite-based composite (Group B) (Cention N, Ivoclar Vivadent, Schaan, Liechtenstein). A split-round Teflon mold was used to create 100 cylindrical samples, with 50 from each material, measuring 10 mm in diameter and 2 mm in thickness. Light polymerization of Beautifil II specimens was performed for 20 seconds using a Mylar strip and glass slab setup, with a 1000 mW/cm² LED curing unit (Bluephase® C8, Ivoclar Vivadent, AG, Schaan, Liechtenstein). To ensure consistent light output (mW/cm2), irradiance was regularly checked with a radiometer from the same manufacturer. For the Cention N specimens, the optimal consistency was achieved by manually mixing two drops of liquid resin with two scoops of powder on a mixing pad. Half of the powder was initially incorporated into the liquid until fully moistened, and the remaining powder was gradually added, ensuring the process was completed within 60 seconds. Then, the prepared paste was evenly spread, placed between a Mylar strip and a glass slab, and packed into the mold with a spatula. Following the manufacturer's instructions, it was left undisturbed to set for two minutes before light curing for 20 seconds with the LED light device (20). Material details are presented in Table 1.

Table 1

**Figure 1:**
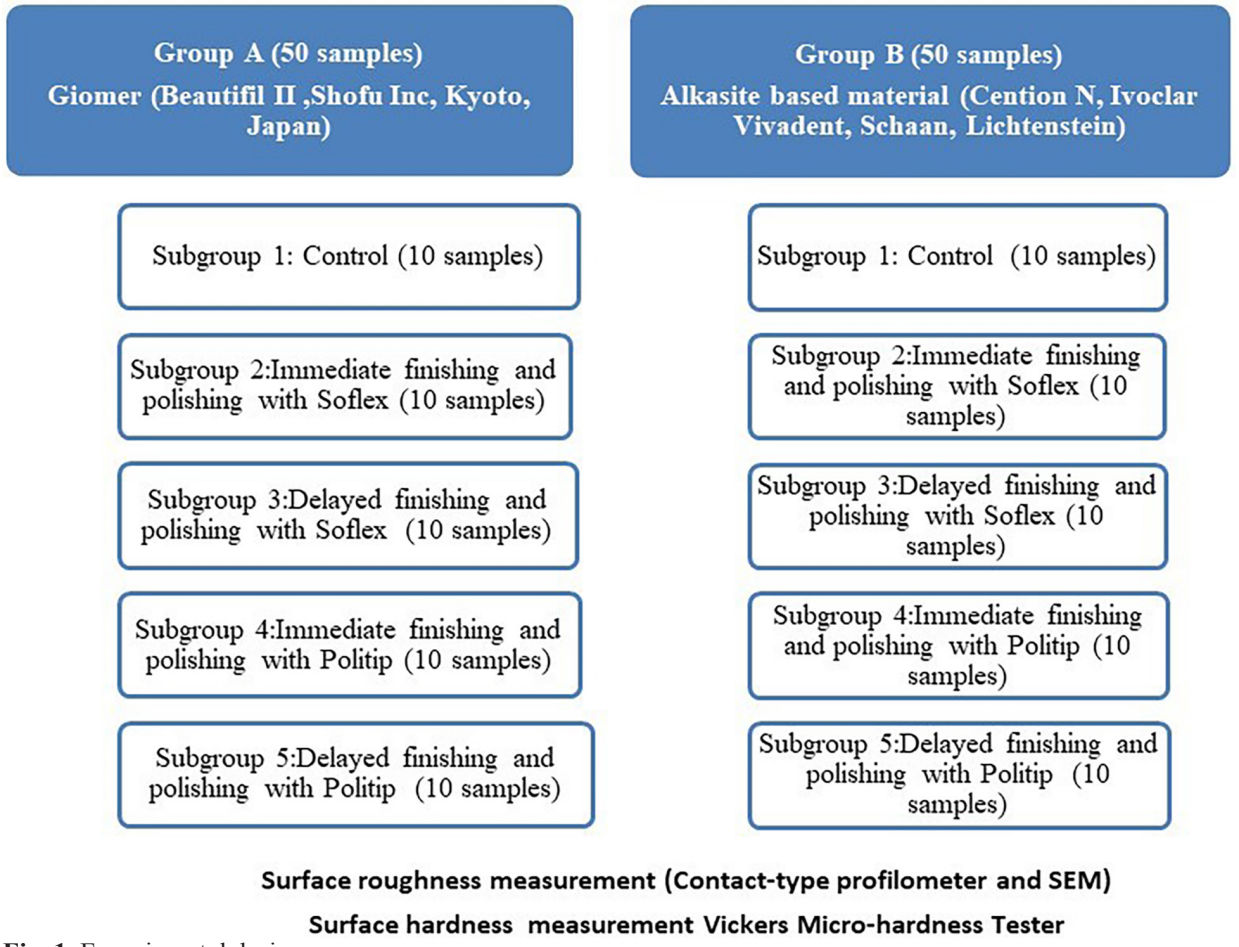
Experimental design.

Once the specimens had set, the discs were demolded and inspected with a magnifying loupe (3× magnification) to identify any visible voids. The experimental layout is shown in Figure 1.

1

Material discs were randomly assigned to one of the finishing subgroups using the F/P technique and timing: Subgroup 1: Serving as the control, ten specimens from each material group were left untreated with any F/P system. Subgroup 2: The discs were finished and polished immediately after material preparation using a multi-step Sof-Lex system. Subgroup 3: The discs were polished and finished using a multi-step Sof-Lex system 24 hours after the materials were prepared. Subgroup 4: Immediately after preparing the materials, the discs were finished and polished with Politip F and Politip P flames. Subgroup 5: The discs were finished and polished with Politip F and Politip P flames 24 hours after the materials were prepared. Details about the polishing systems are shown in Table 1. The finishing and polishing (F/P) process was carried out using a slow-speed handpiece running at 10,000 rpm with water spray and a stopwatch to monitor the time, following the manufacturer's instructions, 10 seconds per step. To maintain consistency across all groups, the operator applied a controlled pressure of 40 grams, recalibrated every ten specimens (19). The specimens were prepared, finished, and polished by a single operator to reduce variability in surface treatment. Specimens in Subgroups 3 and 5 were kept for 24 hours in distilled water before undergoing the F/P process. To remove surface debris after polishing, specimens were rinsed for 20 seconds with distilled water, followed by 5 seconds of air-drying. - Surface roughness measurement A contact-based profilometer (Mitutoyo SJ Series 410, Tokyo, Japan) was used to measure surface roughness. A second operator, unaware of the material type, conducted the evaluations to ensure objectivity. The diamond stylus was intentionally positioned away from the sample edges to prevent potential inaccuracies, as the polishing process might not have evenly affected the margins. For each specimen, surface roughness (Ra) was measured by taking three readings, after rotating the sample by 120 degrees around its center. The profilometer settings included a cutoff length of 0.8 mm, a 4 mm tracing length, and a stylus speed of 0.25 mm/s (21). The average of the three readings was recorded as the final Ra value in micrometers. Calibration of the device was routinely checked throughout the measurement process to ensure accuracy. - Surface Topography Examination Specimens were coated with a conductive silver layer before SEM analysis. Using a focused electron beam, SEM produced high-resolution images of each specimen's surface topography. One specimen from each group was chosen as a representative for SEM analysis. Imaging was performed with a JSM-6510LV SEM (JEOL, USA). Surface images were taken at a magnification of 1000×, offering detailed views of the materials' topographical features (22). - Surface Hardness Assessment Surface microhardness was assessed using a Vickers microhardness apparatus (HVS-50, China). Measurements were performed with a 100 g load applied for 15 seconds. Three indentations were made on each sample, evenly spaced in a circular pattern with no more than 0.5 mm between them (2). The length of the diagonals of these indentations was recorded using the tester's integrated microscope, and the Vickers hardness values were calculated and converted into microhardness values. - Statistical Analysis The Shapiro-Wilk test confirmed the normality of the data distribution. Data analysis was conducted using SPSS software version 17. Two-way ANOVA was used to assess the effects of restorative materials and polishing tools on surface roughness and microhardness. Tukey's post hoc test was performed to identify significant differences between groups.

## Results

The impact of materials and polishing tools on surface roughness is presented in Table 2.

Table 2

Two-way ANOVA revealed a significant interaction between the two factors tested. Additionally, a statistically significant difference was observed between the two restorative materials and F/P systems (P &lt; 0.05). The surface roughness (m) results of the Alkasite and Giomer groups after various finishing protocols are summarized in Table 3.

Table 3

**Figure 2:**
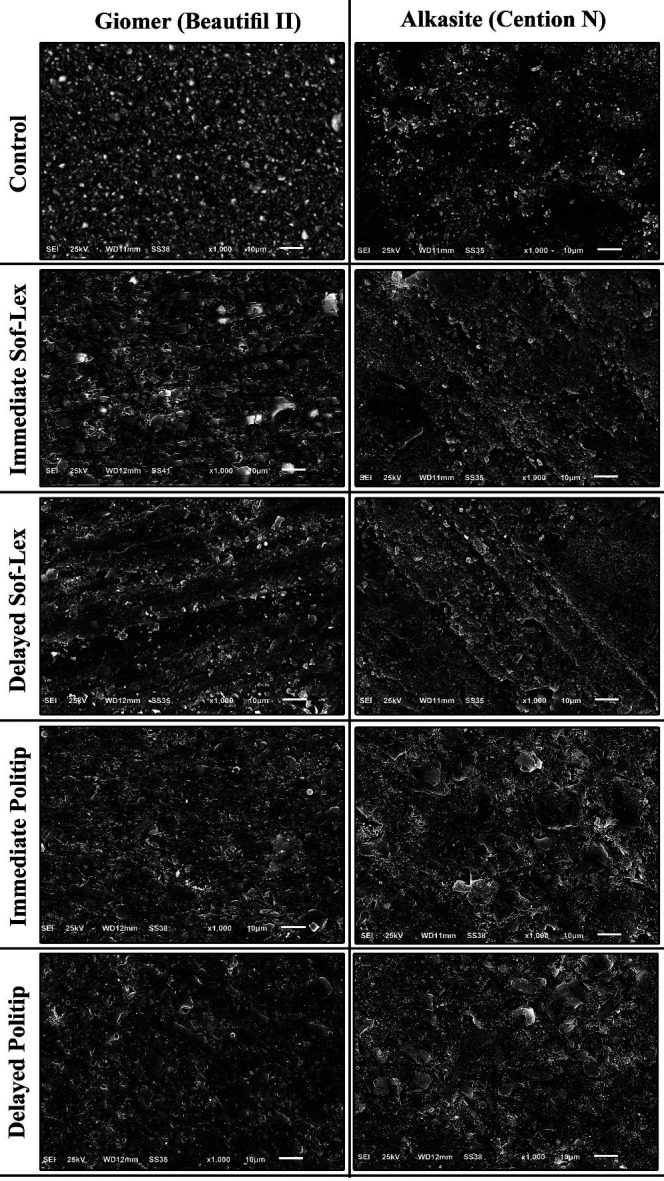
Comparison between the surface topography of Alkasite and Giomer groups.

Alkasite exhibited significantly higher surface roughness values than Giomer. Within the Giomer (A) subgroups, specimens finished with Politip exhibited the highest surface roughness, whether polishing was performed immediately or after 24 hours (p = 0.0024). The recorded values were 0.362 ± 0.082 for the immediate Politip subgroup and 0.360 ± 0.053 for the delayed Politip subgroup. In contrast, there was no significant difference observed between the control subgroup and those finished with Soflex, regardless of whether finishing was done immediately or after 24 hours (p &gt; 0.05). Among Alkasite subgroups, the non-polished specimens showed the lowest mean surface roughness value (0.397±0.260). This difference was statistically significant (p=0.0006). However, there was no significant difference among all polished Alkasite subgroups (P &gt; 0.05). The results for the polished subgroups were: 0.817±0.272, 0.770±0.294, 0.894±0.207, and 0.890±0.288 for the immediate Soflex subgroup, delayed Soflex subgroup, immediate Politip subgroup, and delayed Politip subgroup, respectively. Representative SEM micrographs of all subgroups are shown in Figure 2.

2

SEM analysis indicated that Alkasite did not offer better surface quality compared to Giomer, as Alkasite specimens exhibited more surface irregularities than Giomer. Both Giomer and Alkasite groups experienced greater surface damage when finished with Politip. The effect of materials and polishing systems on microhardness is shown in Table 4.

Table 4

Two-way ANOVA revealed no significant interaction between restorative materials or the polishing method on surface microhardness. Vickers microhardness (HV) results for both Giomer (A) and Alkasite (B) after different finishing protocols were summarized in Table 5.

Table 5

Tukey's post hoc test detected no significant difference in microhardness among Alkasite and Giomer specimens, regardless of F/P systems and timing (P &gt; 0.05).

## Discussion

Achieving a smooth surface and establishing a proper occlusal relationship are crucial for the best clinical outcomes of restorative materials. Therefore, the F/P steps are necessary for successful dental restorations (23). Surface roughness and microhardness are the two most common factors used to compare the surface properties of different restorative materials, as they are related to the aesthetics and functionality of restorations (19). The current study evaluated the effectiveness of different F/P tools and durations on the surface roughness and microhardness of Giomer and Alkasite. In this in vitro experiment, Sof-Lex (multi-step) and Politip (two-step) polishing systems were used to examine the impact of varying application procedures and polishing materials on the surface properties of Giomer and Alkasite. The timing of F/P has a crucial impact on the surface roughness and hardness of restorative materials. The main debate about finishing these materials involves "when to initiate polishing." Polishing time greatly affects the surface roughness and microhardness of resin-based restorations (17). Therefore, in this study, half of the specimens were finished immediately, while the other half were finished after 24 hours to evaluate how polishing time influences these surface properties. Several methods are used to examine the surface roughness of restorative materials, including profilometers, environmental scanning electron microscopes, and atomic force microscopy (2 , 13). A mechanical profilometer was chosen for this study because it measures surface roughness by calculating the average roughness (Ra) value, which is the mean of the vertical deviations of surface irregularities from the central line over the measured sample length (24). To better predict the results, SEM analysis was also conducted to examine surface topography (25). One specimen from each subgroup was analyzed, and representative images of the polished surfaces were captured at 1000× magnification. It should be noted that the control unpolished specimens of both materials displayed lower surface roughness than the polished specimens. This outcome aligns with previous studies reporting that the smoothest surfaces are achieved with Mylar strips, without additional finishing or polishing [11,13.26]. Unpolished Giomer and Alkasite specimens had surface roughness values that did not exceed 0.4 m. These surface roughness values are not clinically detectable when the Ra is below 0.5 m (27). This result may be related to the spherical shape of the fillers in the tested materials, which reflected a smoother surface finish. In contrast, irregular-shaped material fillers produced the roughest surface (28). Removing the superficial layer is still important because the resin-rich oxygen-inhibited layer remains on the smooth, sticky surface (11 , 13). However, finishing and polishing can generate heat through friction, which may cause microcracks in the matrix and increase surface roughness (29). As a result, the polished giomer and alkasite samples showed higher surface roughness values compared to the control group, but still stayed within acceptable limits according to ISO standard No 1302:2002 (30). When compared to Giomer, Alkasite exhibited significantly higher roughness values in both the control and polished samples. This likely results from the larger, harder filler particles in Alkasite, which lead to higher Ra values and more roughness. Additionally, Giomer has a higher inorganic filler content (83% by weight) than Alkasite (78.4% by weight), which could explain its relatively smoother surface. Different finishing and polishing tools have varying effects on the surface roughness of restorative materials (32). Specifically, for resin-based restoratives, surface roughness is mainly affected by the flexibility of the finishing instrument, the hardness of the abrasive material, and the grit size used (13). In this in-vitro study, specimens finished with Soflex discs showed the lowest surface roughness values among all tested samples. Conversely, the highest roughness values were observed in specimens finished and polished with Politip rubber flames. However, there was no significant difference between Soflex disks and Politip in terms of Beautifil Giomer material. Soflex disks are flexible, color-coded, four-step finishing/polishing systems made of elastomer impregnated with aluminum oxide particles. This outcome may be explained by the ability of finishing discs to produce smoother surfaces through planar motion while effectively abrading both the harder filler particles and the softer resin matrix (33). Effective finishing requires abrasives with hardness levels higher than those of the fillers; alternatively, the discs may preferentially abrade the resin matrix, exposing filler particles on the surface (34). The higher roughness seen in specimens finished with Politip rubber flames could be due to their limited ability to uniformly abrade both the fillers and the matrix (13). These findings align with previous studies that identified aluminum oxide-impregnated F/P disks as standard tools for finishing and polishing resin composite restorations (13 , 35). Another parameter evaluated in this study was surface hardness, a key property reflecting resin composites' performance and durability (36 , 37). The findings of this study demonstrated acceptable Vickers hardness values, comparable to the reported microhardness of human dentin (50-60 VHN) (38). Since hardness is indirectly affected by the degree of conversion, all specimens were cured with an LED light in accordance with the manufacturer's guidelines to achieve the necessary degree of conversion (37). Composite hardness is additionally influenced by other factors, including filler particle size, type, shape, distribution, loading, and the composition of the organic matrix (33). In this study, Alkasite and Giomer showed high surface hardness values, likely due to the positive relationship between the materials' inorganic filler content and surface hardness. Giomer contains filler particles averaging 0.8 m in size, with a range from 0.01 to 4.0 m, and a filler loading of 83% by weight (31). The high surface hardness of Alkasite, on the other hand, may be attributed to the filler system consisting of 0.1 m spherical particles. This shape is advantageous because it allows for higher filler incorporation (78.4% by weight) (39). Additionally, the strong filler-matrix bond and the presence of four distinct dimethacrylates (urethane dimethacrylate, tricyclodecandimethanol dimethacrylate, aliphatic-UDMA, and polyethylene glycol dimethacrylate) in the liquid component promote cross-linking during polymerization, further improving its mechanical properties (40). However, no statistical differences in hardness were observed between the specimens finished and polished with either Soflex or Politip. Clinically, resin composites are usually polished and exposed to the oral environment immediately after placement. Previous studies favored delaying the finishing process for at least 24 hours (17). About 75% of the polymerization occurs within the first ten minutes, with the remaining conversion continuing over the next 24 hours if the restoration is stored in water before finishing. Performing finishing right after placement may cause plastic deformation of the material, as heat generated during the process can lead to surface flow (41). The current study's findings showed that immediate polishing did not negatively impact the surface roughness or microhardness of Alkasite compared to delayed polishing. A possible explanation for this result could be the increased filler loading, along with the lower viscosity and greater flexibility of the UDMA monomer in Alkasite, which promote a higher degree of polymerization (42). This outcome might also be due to the minimal thermal insults produced by F/P tools during these F/P procedures when using a water coolant system. The findings are consistent with earlier research indicating that immediate finishing does not harm surface roughness or hardness (43). The microhardness values of Alkasite were similar to those of Giomer, regardless of the finishing/polishing technique or timing. Based on the current results, the timing of finishing and polishing did not significantly affect surface roughness or microhardness of either material. Similarly, the choice of finishing system did not result in significant differences within the subgroups of each material. Therefore, the study's first and second null hypotheses were accepted. The limitation of this study is its in vitro design, which restricts the direct applicability of its findings to clinical conditions without supportive long-term trials to assess the material's performance in practice. Giomer was chosen as the positive control due to its well-documented ability to achieve a highly polished surface, despite not being a bulk-fill material. Future research should compare alkasite with other bulk-fill restoratives and evaluate additional finishing systems and their effects on the surface properties of both Giomer and Cention. Additionally, the study did not include long-term aging or wear simulations, and outcomes such as gloss, color stability, and bacterial adhesion were not assessed. These aspects should be explored in future research to gain a more comprehensive understanding of the materials' clinical performance.

## Conclusions

Given the limitations of this study, neither the timing nor the finishing and polishing system significantly affected the surface roughness or microhardness of Alkasite and Giomer. Both materials exhibited satisfactory surface properties, with no negative impact from immediate compared to delayed polishing. These results indicate that immediate finishing and polishing of Alkasite can be done without harming its surface quality or mechanical performance. Further clinical studies are necessary to confirm these results.

## Figures and Tables

**Table 1:** Materials and finishing systems used in the study.

Material	Manufacturer	Constituents	LOT NO.
Beautifil II restorative	Shofu Inc, Kyoto, Japan	Fluoroboroaluminosilicate glass fillers and nano fillers, Bis-GMA, TEGDMA matrix Camphorquinone	072133
Cention N	Ivoclar Vivadent	ytterbium trifluoride, Br-Al-Si glass fillerIsofiller(copolymer), a calcium barium aluminium fluorosilicate glass filler, UDMA, PEG-400 DMA	Z03CKR
Sof-LexMulti-step F/P system	3M Dental ProductsESPE, St. Paul,Minnesota, UnitedStates	Aluminium OxideCoarse (50-90µmMedium (10-40 µm)Fine (3-9 µm)Superfine (1-7 mm)	N944264
PolitipTwo-step F/PSystem	Ivoclar Vivadent	Rubber flameSilicone rubber, siliconcarbide particles, andtitanium oxide	ZL09G4

Bis-GMA: bisphenol-A-diglycidyl methacrylate; TEGDMA: triethyleneglycol dimethacrylate; UDMA: urethane dimethacrylate; PEG-400 DMA: polyethylene glycol-400 dimethacrylate.

**Table 2:** Two-way ANOVA revealing the impact of restorative material, polishing system, and their interaction on surface roughness (µm).

Variables	Sum of squares	Mean square	F value	P Value
Restorative material	4.81890304	4.81890304	124.26	<.0001
Polishing system	1.37762266	0.34440566	8.88	<.0001
Restorative material* Polishing system	0.45203366	0.11300842	2.91	0.0257
Error	3.49037460	0.03878194		
Corrected Total	10.13893396			

Statistically significant difference at P≤0.05

**Table 3:** Mean surface roughness (µm) and SD of the tested materials.

Subgroup	Group A (Giomer)		Group B (Alkasite)		P value
	Mean	SD	Mean	SD	
1	0.220b	0.027	0.397b	0.260	0.0462
2	0.311ab	0.103	0.817a	0.272	0.0001
3	0.320ab	0.114	0.770a	0.294	0.0003
4	0.362a	0.082	0.894a	0.207	0.0001
5	0.360a	0.053	0.890a	0.288	0.0001
P value	0.0024		0.0006		

Using the Tukey test, means with the same letter in each column are not statistically different at ≤ 0.05.

**Table 4:** Two-way ANOVA revealing the impact of restorative material, polishing system, and their interaction on micro-hardness (Kgf/mm2).

variables	Sum of squares	Mean square	F value	P Value
Restorative material	11.84736400	11.84736400	2.61	0.1100
Polishing system	2.45051000	0.61262750	0.13	0.9691
Restorative material* Polishing system	1.93606600	0.48401650	0.11	0.9800
Error	409.1734600	4.5463718		
Corrected Total	425.4074000			

Statistically significant difference at P≤0.05

**Table 5:** Mean micro-hardness (Kgf/mm2) and SD of the tested materials.

Subgroup	Group A		Group B		P value
	Mean	SD	Mean	SD	
1	90.279a	2.191	91.430 a	2.027	0.2383
2	90.149 a	2.360	90.865 a	2.209	0.4926
3	90.576 a	2.879	90.957 a	2.430	0.7528
4	90.499 a	1.193	90.921 a	1.531	0.5006
5	90.056 a	2.200	90.828 a	1.818	0.4036
P value	0.9822		0.9636		

Using the Tukey test, means with the same letter in each column are not statistically different at ≤ 0.05.

## Data Availability

The datasets used and/or analyzed during the current study are available from the corresponding author.

## References

[1] Jaramillo-Cartagena R, López-Galeano EJ, Latorre-Correa F, Agudelo-Suárez AA (2021). Effect of polishing systems on the surface roughness of nano-hybrid and nano-filling composite resins: A systematic review. Dent J (Basel).

[2] Tepe H, Erdılek AD, Sahın M, Efes BG, Yaman BC (2023). Effect of different polishing systems and speeds on the surface roughness of resin composites. J Conserv Dent.

[3] Pala K, TekÇE N, Tuncer S, SerİM ME, DemİRcİ M (2016). Evaluation of the surface hardness, roughness, gloss and color of composites after different finishing/polishing treatments and thermocycling using a multitechnique approach. Dent Mat J.

[4] Francois P, Fouquet V, Attal JP, Dursun E (2020). Commercially available fluoride-releasing restorative materials: a review and a proposal for classification. Materials (Basel).

[5] Meshram P, Meshram V, Palve D, Patil S, Gade V, Raut A (2019). Comparative evaluation of microleakage around Class V cavities restored with alkasite restorative material with and without bonding agent and flowable composite resin: An in vitro study. Indian J Dent Res.

[6] Naz F, Samad Khan A, Kader MA, Al Gelban LOS, Mousa NMA, Asiri RSH (2021). Comparative evaluation of mechanical and physical properties of a new bulk-fill alkasite with conventional restorative materials. The Saudi dental journal.

[7] François P, Remadi A, Le Goff S, Abdel-Gawad S, Attal JP, Dursun E (2021). Flexural properties and dentin adhesion in recently developed self-adhesive bulk-fill materials. Journal of oral science.

[8] Ruengrungsom C, Burrow MF, Parashos P, Palamara JE (2020). Evaluation of F, Ca, and P release and microhardness of eleven ion-leaching restorative materials and the recharge efficacy using a new Ca/P containing fluoride varnish. J Dent.

[9] Kozmos M, Virant P, Rojko F, Abram A, Rudolf R, Raspor P (2021). Bacterial adhesion of Streptococcus mutans to dental material surfaces. Molecules.

[10] Bollen C, Lambrechts P, Quirynen M (1997). The surface roughness of different oral hard materials in comparison to the threshold surface roughness for bacterial plaque retention. A review of the literature. Dent Mater.

[11] Pietrokovski Y, Zeituni D, Schwartz A, Beyth N (2022). Comparison of Different Finishing and Polishing Systems on Surface Roughness and Bacterial Adhesion of Resin Composite. Materials.

[12] Kemaloglu H, Karacolak G, Turkun LS (2017). Can reduced-step polishers be as effective as multiple-step polishers in enhancing surface smoothness?. J Esthet Restor Dent.

[13] Soliman HA, Elkholany NR, Hamama HH, El-Sharkawy FM, Mahmoud SH, Comisi JC (2020). Effect of different polishing systems on the surface roughness and gloss of novel nanohybrid resin composites. Eur J Dent.

[14] Samuel A, Raju R, Sreejith K, Kalathil BM, Nenavath D, Chaitra V (2020). Comparative evaluation of the surface hardness of different esthetic restorative materials: An in vitro study. J Pharm Bioallied Sci.

[15] Lins FCR, Ferreira RC, Silveira RR, Pereira CNB, Moreira AN, Magalhães CS (2016). Surface roughness, microhardness, and microleakage of a silorane-based composite resin after immediate or delayed finishing/polishing. Int J Dent.

[16] Ghasemi A, Mohammadzadeh A, Molaei M, Sheikh-Al-Eslamian SM, Karimi M (2023). Effect of Wet and Dry Finishing and Polishing Technique on Microhardness and Flexural Strength of Nanocomposite Resins. International Journal of Dentistry.

[17] Madhyastha PS, Hegde S, Srikant N, Kotian R, Iyer SS (2017). Effect of finishing/polishing techniques and time on surface roughness of esthetic restorative materials. Dent Res J.

[18] Vishwanath S, Kadandale S, kumar Kumarappan S, Ramachandran A, Unnikrishnan M, manjiri Nagesh H (2022). Finishing and polishing of composite restoration: assessment of knowledge, attitude and practice among various dental professionals in India. Cureus.

[19] Carrillo-Marcos A, Salazar-Correa G, Castro-Ramirez L, Ladera-Castañeda M, López-Gurreonero C, Cachay-Criado H (2022). The microhardness and surface roughness assessment of bulk-fill resin composites treated with and without the application of an oxygen-inhibited layer and a polishing system: an in vitro study. Polymers.

[20] Bahari M, Kahnamoui MA, Chaharom MEE, Kimyai S, Sattari Z (2021). Effect of curing method and thermocycling on flexural strength and microhardness of a new composite resin with alkaline filler. Dental research journal.

[21] Setty A, Nagesh J, Marigowda JC, Shivanna AK, Paluvary SK, Ashwathappa GS (2019). Comparative Evaluation of Surface Roughness of Novel Resin Composite Cention N With Filtek Z350 XT: In Vitro: Study. International Journal of Oral Care and Research.

[22] Kılıç V, Gök A (2021). Effect of different polishing systems on the surface roughness of various bulk-fill and nano-filled resin-based composites: An atomic force microscopy and scanning electron microscopy study. Microscopy Research and Technique.

[23] Ismail HS, Ali AI, El-Ella MAA, Mahmoud SH (2020). Effect of different polishing techniques on surface roughness and bacterial adhesion of three glass ionomer-based restorative materials: In vitro study. J Clin and Exp Dent.

[24] Wheeler J, Deb S, Millar BJ (2020). Evaluation of the effects of polishing systems on surface roughness and morphology of dental composite resin. Br D J.

[25] Oglakci B, Kucukyildirim B, Özduman Z, Eliguzeloglu Dalkilic E (2021). The effect of different polishing systems on the surface roughness of nanocomposites: Contact profilometry and SEM analyses. Oper Dent.

[26] Zhang L, Yu P (2021). Surface roughness and gloss of polished nanofilled and nanohybrid resin composites. J Dent Sci.

[27] Jones CS, Billington RW, Pearson GJ (2004). The in vivo perception of roughness of restorations. Br Dent J.

[28] Ruivo MA, Pacheco RR, Sebold M, Giannini M (2019). Surface roughness and filler particles characterization of resin-based composites. Micro Res Tech.

[29] Khabadze Z, Ivanov S, Kotelnikova A, Protsky M, Dashtieva M (2021). The influence of finishing processing features on the polymerized composite surface structure. Georgian Medical News.

[30] (2002). Specifications GP. Indication of surface texture in technical product documentation.

[31] Gömleksiz S, Gömleksiz O (2022). The effect of contemporary finishing and polishing systems on the surface roughness of bulk fill resin composite and nanocomposites. J Esthet Restor Dent.

[32] Nithya K, Sridevi K, Keerthi V, Ravishankar P (2020). Evaluation of surface roughness, hardness, and gloss of composites after three different finishing and polishing techniques: an in vitro study. Cureus.

[33] St-Pierre L, Martel C, Crépeau H, Vargas M (2019). Influence of polishing systems on surface roughness of composite resins: polishability of composite resins. Oper Dent.

[34] Sang EJ, Chung SH (2021). Influence of a new polishing system on changes in gloss and surface roughness of resin composites after polishing and brushing. Dent Mat J.

[35] Shen C, Rawls HR, Esquivel-Upshaw JF (2021). Phillips’ Science of Dental Materials E-Book. Elsevier Health Sciences.

[36] Yılmaz Atalı P, Doğu Kaya B, Manav Özen A, Tarçın B, Şenol AA, Tüter Bayraktar E (2022). Assessment of micro-hardness, degree of conversion, and flexural strength for single-shade universal resin composites. Polymers.

[37] Szczesio-Wlodarczyk A, Domarecka M, Kopacz K, Sokolowski J, Bociong K (2021). An evaluation of the properties of urethane dimethacrylate-based dental resins. Materials.

[38] Yazkan B, Celik E, Recen D (2021). Effect of Aging on Surface roughness and color Stability of a novel alkasite in comparison with current direct restorative materials. Oper Dent.

[39] Verma V, Mathur S, Sachdev V, Singh D (2020). Evaluation of compressive strength, shear bond strength, and microhardness values of glass-ionomer cement Type IX and Cention N. J Conserv Dent.

[40] Sardana A, Kumar M, Taneja S (2022). Comparative evaluation of microleakage and hardness of newer posterior restorative materials. J Oral Biol Craniofac Res.

[41] Monterubbianesi R, Tosco V, Sabbatini S, Orilisi G, Conti C, Özcan M (2020). How can different polishing timing influence methacrylate and dimethacrylate bulk-fill composites?. Evaluation of chemical and physical properties. BioMed Res Int.

[42] Kaptan A, Oznurhan F, Candan M (2023). In Vitro Comparison of Surface Roughness, Flexural, and Microtensile Strength of Various Glass-Ionomer-Based Materials and a New Alkasite Restorative Material. Polymers.

[43] Yazici AR, Tuncer D, Antonson S, Onen A, Kilinc E (2010). Effects of delayed finishing/polishing on surface roughness, hardness and gloss of tooth-coloured restorative materials. Eur J Dent.

